# Temporal Baseline of Essesntial and Non-essential Elements Recorded in Baleen of Western Arctic Bowhead Whale (*Balaena mysticetus*)

**DOI:** 10.1007/s00128-021-03394-2

**Published:** 2021-11-12

**Authors:** Samantha L. Shore, Dimitrios G. Giarikos, Lawrence K. Duffy, Mickie R. Edwards, Amy C. Hirons

**Affiliations:** 1 Department of Marine and Environmental Sciences, Halmos College of Arts and Sciences, Environmental Conservation through Leading-Edge Research (SECLER), Nova Southeastern University, 8000 North Ocean Drive, Dania Beach, FL 33004 USA; 2 Department of Chemistry and Physics, Halmos College of Arts and Sciences, Nova Southeastern University, 3301 College Avenue, Fort Lauderdale, FL 33314 USA; 3 Department of Chemistry & Biochemistry, University of Alaska Fairbanks, 1930 Yukon Drive Rm. 194, Fairbanks, AK 99775 USA

**Keywords:** Elements, Metals, Bowhead whale, Baleen, Arctic, SECLER

## Abstract

This study established the first baseline of changing elemental concentrations in bowhead whale baleen over time (1958–1999). From previously published stable isotope data, year, season (summer or winter), and location (Beaufort or Bering/Chukchi seas) were attributed to each sample. Thirteen elements (Al, Cd, Co, Cr, Cu, Fe, Hg, Mn, Ni, Pb, Se, V, Zn) in baleen from nine subsistence-harvested bowhead whales (n = 138) were detected. Al, Cu, and Fe were the highest concentrations while Cd and V were among the lowest. Our data supports absorption as the main route of exposure to environmental elements rather than biomagnification due to bowhead whales’ low trophic position. A linear mixed-effects model confirmed most elements’ concentrations increased with time, while location and sex were insignificant explanatory factors. These temporal fluctuations were most likely a product of environmental changes due to a warming climate and human activities.

**Supplementary Information:**

The online version contains supplementary material available at 10.1007/s00128-021-03394-2.

Human activities (mining, industrial pollution, climate change) have caused environmental concentrations of many elements to increase (Rydberg et al. 2010; CRS 2020). As marine organisms move through various bodies of water and forage, element uptake is dependent upon availability and dietary intake (Tchounwou et al. 2003). This has sparked concern regarding the potential toxic effects of marine life, especially in species of subsistence and commercial importance. Western Arctic bowhead whales (*Balaena mysticetus*) migrate among the Bering, Chukchi, and Beaufort seas, and have been relied upon for centuries by Russian and Alaskan native communities, providing sustenance and other resources (Schell et al. 1989).

Bowhead whales are valuable bioindicators of environmental changes due to their long-life span and ability to biologically incorporate elements from diet and surrounding water (Das et al. 2003; Vos et al. 2003). These whales have baleen plates that are metabolically inert and continuously growing. Elements from the planktonic rich diet are incorporated into the keratinous matrix. Adult bowhead whales have plates of up to 4 m in length and representing 20+ years of growth, thus providing a timeline of environmental elemental concentrations (Schell et al. 1989; Pomerleau et al. 2018). We determined the elemental concentrations of baleen from western Arctic bowhead whales across space and time. Schell et al. (1989) used stable carbon and nitrogen isotope ratios along the whales’ baleen plates to corroborate locations along the migration route with locations along the length of the plates. This allowed them to determine seasonality and annual periodicity based on the whales’ migration patterns.

The analysis of baleen for element concentrations provides a novel method for assessing environmental conditions at specific locations and times. In such a rapidly changing Arctic environment, it is critical to establish temporal elemental concentrations in Arctic wildlife. This information can track environmental changes to help monitor Arctic marine ecosystems.

## Materials and Methods

Baleen samples from nine bowhead whales were obtained from the Alaska Stable Isotope Facility at the University of Alaska Fairbanks where stored. All baleen plates were initially collected during subsistence hunts in the villages of Barrow (Utqiaġvik) and Kaktovik, Alaska. Baleen samples were collected by Schell et al. (1989) at 5 cm intervals from base to tip using a Dremel and tungsten carbide boring tip. Carbon-13 and nitrogen-15 analysis revealed oscillating patterns of enrichment and depletion that the Schell team ascribed to isotopic differences in locations (Bering/Chukchi vs Beaufort seas). There was no isotopic distinction between Bering and Chukchi seas so they were considered the same location. These bodies of water are the terminal ends of the bowhead whale migration route. As bowhead whales migrate seasonally (biannually) between these seas, seasons were defined, summer in the Beaufort Sea and winter in the Bering/Chukchi seas. The compilation of all sample points along the baleen plate produced isotopic patterns corresponding to the primary and secondary production between the respective seas. The distinct stable isotope differences between these two bodies of water served as markers for Schell et al. to calculate the time between seasons (winter/summer) and locations. These isotopic patterns consistently repeat along the baleen plate for all the bowhead whales. We used the remaining samples from Schell’s original baleen plates and their calculated timeline and locations to assign elemental concentrations through space and time. Two sequential baleen samples, representing specific location and season, were combined to achieve optimal mass for elemental analysis (minimum 0.1 g). It should be noted that samples were taken from sub-adult and adult whales, some of which had a noted body length (Supplemental Table 1). A total of 138 samples across nine whales were analyzed and represented a minimum of one sample per year from 1958 to 1999 (except 1967–1968). This yielded ~ 41 years of seasonal (winter, summer) and locational elemental data for the western Arctic bowhead whale population.

Samples were digested with Trace Metal Grade nitric acid in 100 mL polypropylene digestion tubes, then placed in a ModBlock at 60°C for 24 h until samples were entirely digested. Each sample was diluted to 5% nitric acid or less with ultrapure deionized water (18.2 MΩ). The concentrations of 13 elements—Al, Cd, Co, Cr, Cu, Fe, Hg, Mn, Ni, Pb, Se, V, Zn were analyzed at the University of Southern Mississippi Center for Trace Analysis via sector-field inductively coupled plasma mass spectrometry (ICP-MS, ThermoFisher Element XR) with a Peltier-cooler spray chamber (PC-3; Elemental Scientific, Inc.). Blanks of ultrapure deionized water and trace metal basis nitric acid (3%, 4%, 5%) were used for quality control. The ICP-MS detection limits of each element are provided in Supplemental Table 2. Human hair certified reference material (Sigma Aldrich ERMDB001) was used to test our dissolution method. Cd, Cu, Se, and Zn concentration efficiencies were 92%, 115%, 86%, and 96%, respectively. Hg (61%) was relatively low compared to the certified study efficiency, likely due to some Hg adsorption by our plastic polypropylene digestion tubes. The relatively higher concentration of Pb detected could be due to contamination though none was readily identified.

All statistical analyses were conducted in the open-source software R Studio (License AGPL v3). A linear mixed-effects model (LMM) with Gaussian distribution and identity link functions was applied to each element to test for the significance of location, sex, and time (year), and/or their interaction term on concentration (Supplemental Table 3). Age and growth rate could not be included in the models due to limited sample data; this prevented intraspecific statistical comparisons. Instead, a linear mixed-effects model was applied to each whale’s samples separately from the other whales. Each LMM was fit by restricted maximum likelihood through the function lme in the R package nlme. All models had whale ID as a random effect to account for repeated measures of each response variable along the length of each baleen plate. A backwards-step selection with the help of Akaike’s information criterion (AIC) model selection finalized the best-fit model. The final model, selected based on the lowest AIC score, included year and whale, but not location and sex, for all 13 elements. The final model was represented by: (element ~ year, random =  ~ 1|whale, method = "REML"), where element was the element under investigation (Supplemental Table 4).

## Results and Discussion

All 13 elements (Al, Cd, Co, Cr, Cu, Fe, Hg, Mn, Ni, Pb, Se, V, Zn) were detected in bowhead whale baleen (Supplemental Table 5). Al, Cu, and Fe had the highest elemental concentrations, reaching into the thousands of µg/g (Supplemental Fig. 1a, e, f). Cd, Hg, and V concentrations were the lowest compared to all other elements, with concentrations below 10 µg/g (Supplemental Fig. 1b, g, l). The detected concentrations were likely influenced by varying ecological and environmental factors for each whale used in this study. The exact migratory paths of each whale were unknown, though Schell et al.’s stable isotope data indicated that each whale migrated annually between the Bering/Chukchi seas and Beaufort Sea. The whales sampled here also differed in age, physiological status, and body length (Supplemental Table 1).

Al, Cu, Fe, and Zn had high standard deviations likely due to the high variance in the data and low sample size per year (Shore 2020, Tables 4 and 5). Al, Fe, and Zn increased over time with higher concentrations in the mid to late 1990s (1995–1999). The highest concentrations of all elements, excluding Cu, were found mostly in whales hunted between 1995 and 1999. Cu did not increase over time but did have sporadic high concentrations (Supplemental Fig. 1e).

Environmental elements likely accumulated in baleen through various routes. In marine mammals, elements mainly enter and accumulate in the body through ingestion (Becker et al. 1997; Das et al. 2003; Vos et al. 2003). The main food source for bowhead whales is zooplankton, largely occupying a trophic position of 2.0. Bowhead whales have a lower trophic position (3.20) compared to other marine mammal species (3.40–4.70) due to the prey/dietary differences. Elemental biomagnification at low trophic levels will likely have a nominal effect on concentration increases (Trites 2019). St. Aubin et al. (1984) found naturally existing Cu, Fe, and Zn in bowhead whale baleen (means 8, 20, and 216 µg/g, respectively) but no Mn. Campbell et al. (2005) found Cu, Zn, and Cd concentrations in Arctic zooplankton in Baffin Bay (1.5–1.6, 14–17, and 0.9–1.6 µg/g, respectively). Our study yielded Cu and Zn concentrations (8–1400 and 198–1600 µg/g, respectively), 5 to 1000 times higher in bowhead whale baleen, while our Cd concentrations (0.02–3.5 µg/g) were comparable to the zooplankton. Mn, Pb, and Se concentrations in zooplankton (0.3–0.4, 0.3–1, and 1.8–1.9 µg/g, respectively) were similar to those in our bowhead whale baleen (0.3–80, 0.7–200, and 0.4–3.4 µg/g respectively) prior to the mid 1990s (Supplemental Table 5). The LMMs (Supplemental Tables 3 and 4) showed that eight of the 13 elements tested (Al, Cd, Cr, Fe, Mn, Ni, Pb, Zn) significantly increased over time across all nine whales with the highest concentrations in baleen samples representing the 1990s (Supplemental Fig. 1a, b, d, f, h, i, j, m). Cu and Se concentrations were the only elements to exhibit their highest values prior to 1966 (Supplemental Fig. 1e, k).

Biosorption of elements from the surrounding water through adhesion and absorption are supported as the dominant mechanism. Baleen is made of hard keratin, which has been proven absorptive, especially when hydrated (Kar and Misra 2004; Werth et al. 2016; Donner et al. 2019). Baleen naturally contains 34.37% water but when hydrated, stores excess water (Werth et al. 2016). The absorption mechanism of trace elements has not been studied to our knowledge, though several studies have utilized keratinous structures, such as avian feathers. Because feathers and baleen are both hard keratins, they likely share similar absorption mechanisms. Co, Cr, Cu, Hg, Pb, Ni, and Zn were successfully absorbed from water into feathers, though pH did influence uptake (Kar and Misra 2004; Donner et al. 2019). Keratin absorbs metal ions through both physiosorption and chemiosorption. Physiosorption, such as wet adhesion, traps metal ions in nanostructured surfaces, like the nanoporous surface of baleen. Chemiosorption occurs at binding sites of keratin (Kar and Misra 2004). Further research utilizing baleen as a biosorbent is needed to understand how environmental concentrations adhere, adsorb, or absorb in baleen.

The Arctic environment has been warming at an unprecedented rate and the Arctic Ocean is especially sensitive to these changes. Higher atmospheric temperatures lead to ice and permafrost melting, riverine discharge, and coastal erosion (Overland et al. 2002; Rydberg et al. 2010; Jensen et al. 2021). Snow, ice, and permafrost store and accumulate trace elements over time, then release large concentrations into the marine Arctic environment through melting events and runoff. As riverine output intensifies, these waters carry higher concentrations of elements towards the coastal environment. (Perryman et al. 2020; Polyakov et al. 2010; Tovar-Sánchez et al. 2010; Schaefer et al. 2020). Rember and Trefry (2004) found that trace metal (Fe, Cu, Pb, Zn) concentrations in Alaskan rivers were highest during deluges from snowmelt and erosion of upper soil layers. Ocean warming changes gas solubility and pH concentrations and the resulting acidification increases the concentrations and bioavailability of metal ions such as Fe, Cu, Zn, and Cd (Millero et al. 2009; Avendaño et al. 2016; Lewis et al. 2016).

All nine whales had some of their highest elemental concentrations in a single baleen sub-sample. For example, whale 90B8 had its highest concentrations of As, Cr, Fe, Mn, Ni, and Se in summer 1981 (Beaufort Sea), while Cd, Co, Cu, Hg, Pb, and V concentrations were highest in summer 1979 (Beaufort Sea). In addition, samples from different whales that overlapped in time and/or location had similar elemental concentration increases. Whale 95B8 and 99KK1 had their highest concentrations of Al, Co, Cr, Cu, Fe, Hg, Mn, Ni, and Zn in summer 1995 (Beaufort Sea). The five whales hunted in the 1990s had concentrations of Fe, Mn, and Ni that covaried (similar increases and decreases) across the entire baleen plate Whales 97B8, 98KK1, and 99KK1 had Fe and Mn concentrations that covaried similarly across the entire baleen plate. Whales 90B8 and 95B8 had Fe, Mn, and Ni concentrations that changed similarly with time across the baleen plate (Supplemental Fig. 1f, h, i). It is known that Fe and Mn share many common sources and sinks in the global ocean (Jensen et al. 2020). Data showed that Se and Hg concentrations were not significantly correlated (*p* > 0.05). Se concentrations decreased over time while Hg concentrations were highest in whales 66B1 and 78B2 and whales landed in the 1990s (Supplemental Fig. 1 g, k). An increase in environmental Hg from 1990 to 2000 was discovered polar bears, beluga whales, and ringed seals (Wagemann et al. 1996; Woshner et al. 2001; Kannan et al. 2007). Therefore, we surmised the Hg increase during the 1990s was likely due to environmental increases that other Arctic species experienced. With no anomalous temperatures or precipitation events in the years the samples represented, it is believed that short-lived environmental fluxes were the cause of these synchronous high concentrations. Since the Mackenzie River influences the chemistry of the Beaufort Sea and surface waters in the Western Arctic, atmospheric warming and subsequent melt events may contribute to these intermittent recordings (Macdonald et al. 2000; Carmack et al. 2016).

Additional data are needed to further our understanding of the effect of a changing Arctic on the marine environment and how marine mammals physiologically respond to changing elemental concentrations. Because Arctic marine mammals play a critical role in the ecological function of marine ecosystems and are often relied upon by indigenous communities, understanding these relationships are critical, particularly as the climate and ocean chemistry continue to fluctuate.

## Supplementary Information

Below is the link to the electronic supplementary material.

Supplementary file1 (PDF 413 kb)

## References

[CR1] Avendaño L, Gledhill M, Achterberg EP, Rérolle VMC, Schlosser C (2016). Influence of ocean acidification on the organic complexation of iron and copper in northwest European shelf seas; a combined observational and model study. Front Mar Sci.

[CR30] Becker PR, Mackey EA, Demiralp R, Koster BJ, Wise SA (1997) Establishing baseline levels of elements in marine mammals through analysis of banked liver tissues. In: Environmental monitoring and specimen banking, ACS Symposium Series 654. American Chemical Society, Washington, D.C

[CR2] Campbell LM, Norstrom RJ, Hobson KA (2005). Mercury and other trace elements in a pelagic Arctic marine food web (Northwater Polynya, Baffin Bay). Sci Tot Environ.

[CR31] Carmack EC, Yamamoto-Kawai M, Haine TWN, Bacon S, Bluhm BA, Lique C, Melling H, Polyakov IV, Straneo F, Timmermans M-L (2016). Freshwater and its role in the Arctic Marine System: sources, disposition, storage, export, and physical and biogeochemical consequences in the Arctic and global oceans. J Geophys Res Biogeosci.

[CR32] Congressional Research Service (CRS) (2020) Changes in the Arctic: background and issues for congress. CRS Report R41153

[CR3] Das K, Debacker V, Pillet S, Bouquegneau JM (2003). Heavy metals in marine mammals.

[CR4] Donner MW, Arshad M, Ullah A, Siddique T (2019). Unravelled keratin-derived biopolymers as novel biosorbents for the simultaneous removal of multiple trace metals from industrial wastewater. Sci Tot Environ.

[CR7] Jensen LT, Morton P, Twining BS, Heller MI, Hatta M, Measures CI, John S, Zhang R, Pinedo-Gonzalez P, Sherrell RM, Fitzsimmons JN (2020). A comparison of marine Fe and Mn cycling: U.S. GEOTRACES GN01 Western Arctic case study. Geochim Cosmochim Acta.

[CR8] Jensen LT, Lanning NT, Marsay CM et al (2021) Biogeochemical cycling of colloidal trace metals in the Arctic cryosphere. J Geophys Res Oceans 126 10.1029/2021JC017394

[CR9] Kannan K, Agusa T, Evans TJ, Tanabe S (2007). Trace element concentrations in livers of polar bears from two populations in northern and western Alaska. Arch Environ Contam Toxicol.

[CR10] Kar S, Misra M (2004). Use of keratin fiber for separation of heavy metals from water. J Chem Technol Biot.

[CR12] Lewis CL, Ellis RP, Vernon V, Elliot K, Newbatt S, Wilson RW (2016). Ocean acidification increases copper toxicity differentially in two key marine invertebrates with distinct acid-base responses. Sci Rep.

[CR33] Macdonald RW, Barrie LA, Bidleman TF (2000). Contaminants in the Canadian Arctic: 5 years of progress in understanding sources, occurrence and pathways. Sci Total Environ.

[CR13] Millero FJ, Woosley R, DiTrolio B, Waters J (2009). Effect of ocean acidification on the speciation of metals in seawater. Oceanography.

[CR14] Overland JE, Wang M, Bond NA (2002). Recent temperature changes in the Western Arctic during spring. J Clim.

[CR15] Pomerleau C, Matthews CJD, Gobell C (2018). Mercury and stable isotope cycles in baleen plates are consistent with year-round feeding in two bowhead whale (*Balaena mysticetus*) populations. Polar Biol.

[CR16] Perryman CR, Wirsing J, Bennett KA (2020). Heavy metals in the Arctic: distribution and enrichment of five metals in Alaskan soils. PLoS ONE.

[CR17] Polyakov IV, Timokhov LA, Alexeev VA (2010). Arctic ocean waring contributes to reduced polar ice cap. J Phys Oceanogr.

[CR34] Rember RD, Trefry JH (2004). Increased concentrations of dissolved trace metals and organic carbon during snowmelt in rivers of the Alaskan arctic. Geochim Cosmochim Acta.

[CR18] Rydberg J, Klaminder J, Rosen P, Bindler R (2010). Climate driven release of carbon and mercury from permafrost mires increases mercury loading to sub-arctic lakes. Sci Total Environ.

[CR19] Schaefer K, Elshorbany Y, Jafarov E (2020). Potential impacts of mercury released from thawing permafrost. Nat Commun.

[CR20] Schell DM, Saupe SM, Haubenstock N, Rundel PW, Ehleringer JR, Nagy KA (1989). Natural isotope abundances in bowhead whale (*Balaena mysticetus)* baleen: markers of aging and habitat usage. Stable isotopes in ecological research. Ecological studies (analysis and synthesis).

[CR21] Shore SL (2020) Spatial and temporal distribution of essential and non-essential elements recorded in western arctic bowhead whales (*Balaena mysticetus*). Master's thesis. Nova Southeastern University. https://nsuworks.nova.edu/occ_stuetd/525

[CR22] St. Aubin DJ, Stinson RG, Geraci JR (1984). Aspects of the structure and composition of baleen, and some effects of exposure to petroleum hydrocarbons. Can J Zool.

[CR23] Tchounwou PB, Ayensu WK, Ninashvilli N, Sutton D (2003). Environmental exposures to mercury and its toxicopathologic implications for public health. Environ Toxicol.

[CR24] Tovar-Sánchez A, Duarte CM, Alonso JC, Lacorte S, Tauler R, Galbán-Malagón C (2010). Impacts of metals and nutrients released from melting multiyear Arctic sea ice. J Geophys Res.

[CR25] Trites AW, Cochran JK, Bokuniewicz H, Yager P (2019). Marine mammal trophic levels and interactions. Encyclopedia of ocean sciences.

[CR26] Vos JG, Bossart GD, Fournier M, O’Shea TJ (2003). Toxicology of marine mammals.

[CR27] Wagemann R, Innes S, Richard PR (1996). Overview and regional and temporal differences of heavy metals in Arctic whales and ringed seals in the Canadian Arctic. Sci Tot Environ.

[CR35] Werth AJ, Harriss RW, Rosario MV (2016). Hydration affects the physical and mechanical properties of baleen tissue. R Soc Open Sci.

[CR29] Woshner VM, O’Hara TM, Bratton GR, Suydam RS, Beasley VR (2001). Concentrations and interactions of selected essential and non-essential elements in bowhead and beluga whales of Arctic Alaska. J Wildl Dis.

