# Supplementary material for: Temporal Baseline of Essesntial and Non-essential Elements Recorded in Baleen of Western Arctic Bowhead Whale (Balaena mysticetus)
Source: Bull Environ Contam Toxicol. 2021 Nov 12;108(4):641–5. doi: 10.1007/s00128-021-03394-2 (PMC8979895; doi:10.1007/s00128-021-03394-2)
Supplement: Supplementary file 1 — Supplementary file1 (PDF 413 kb) [file 128_2021_3394_MOESM1_ESM.pdf]

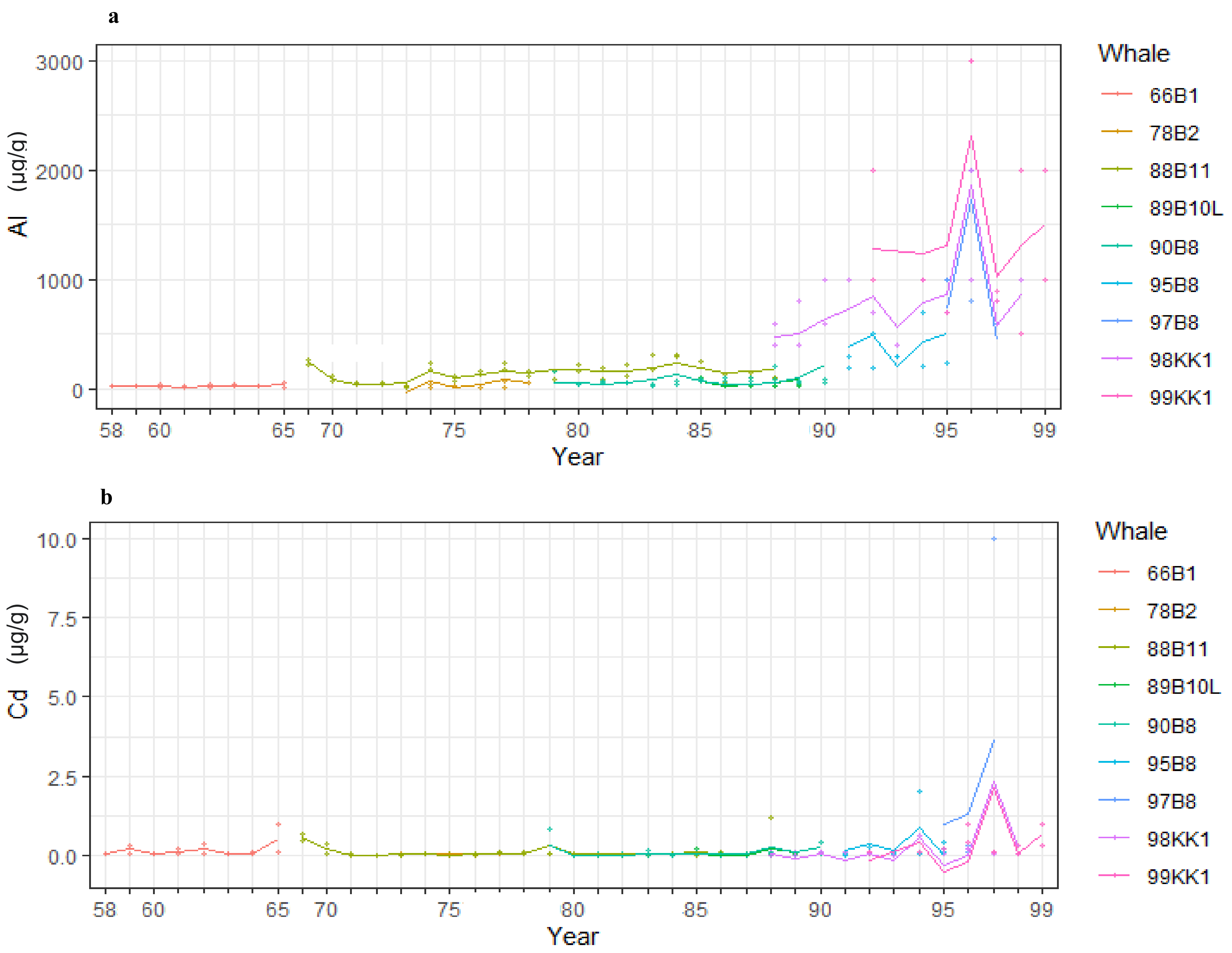
a
Al (µg/g)
3000
2000
1000
0
58 60 65 70 75 80 85 90 95 99
Year
Whale
66B1
78B2
88B11
89B10L
90B8
95B8
97B8
98KK1
99KK1
b
Cd (µg/g)
10.0
7.5
5.0
2.5
0.0
58 60 65 70 75 80 85 90 95 99
Year
Whale
66B1
78B2
88B11
89B10L
90B8
95B8
97B8
98KK1
99KK1

c

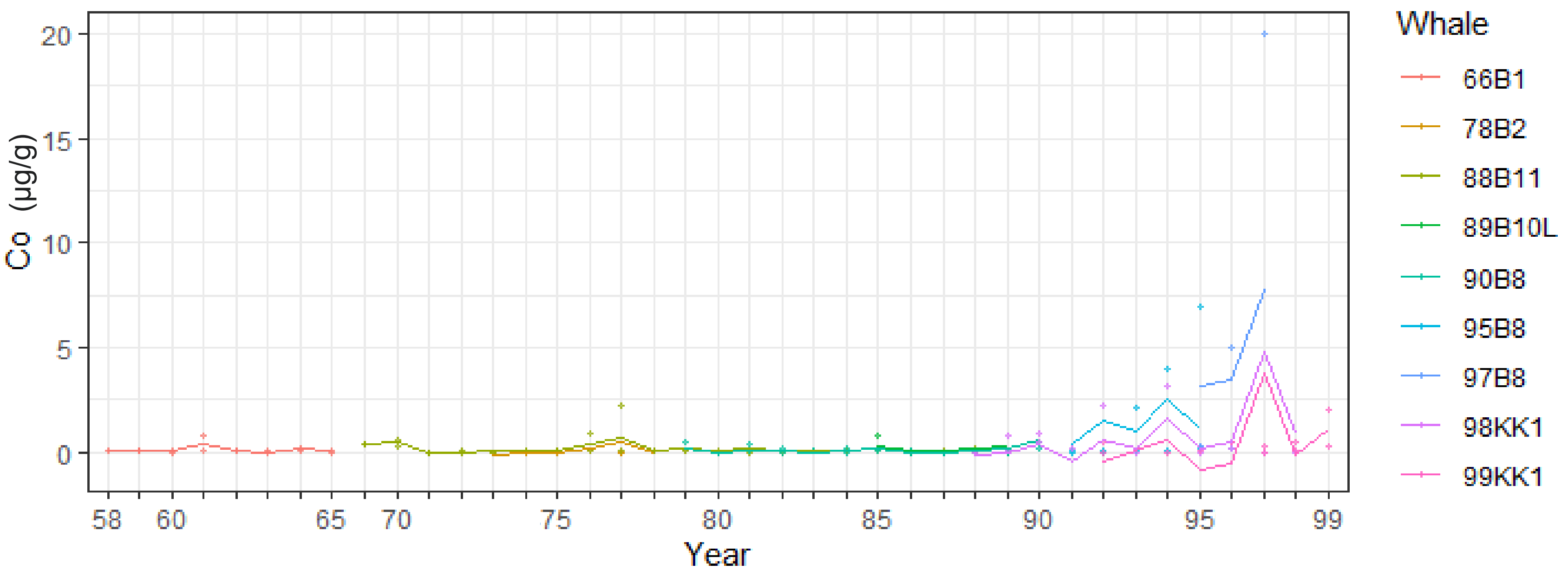

Co (µg/g)
Year
Whale
66B1
78B2
88B11
89B10L
90B8
95B8
97B8
98KK1
99KK1

d

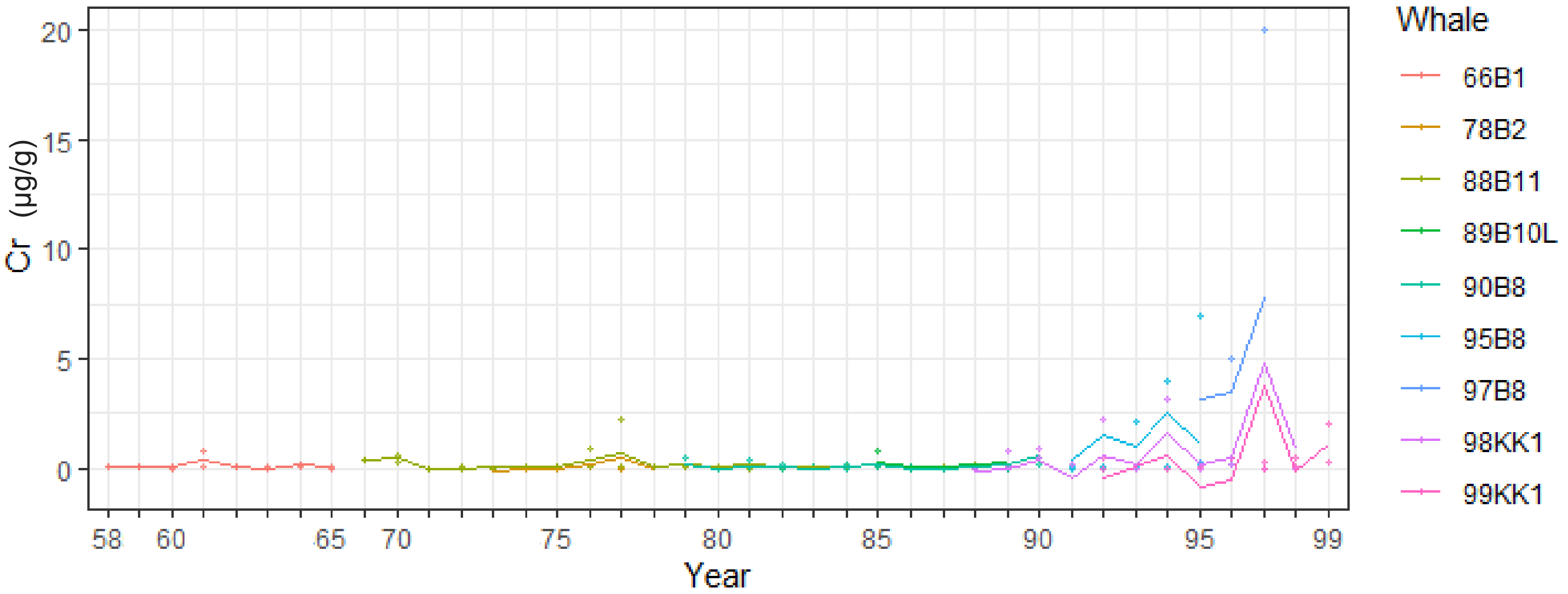

Cr (µg/g)
Year
Whale
66B1
78B2
88B11
89B10L
90B8
95B8
97B8
98KK1
99KK1

e

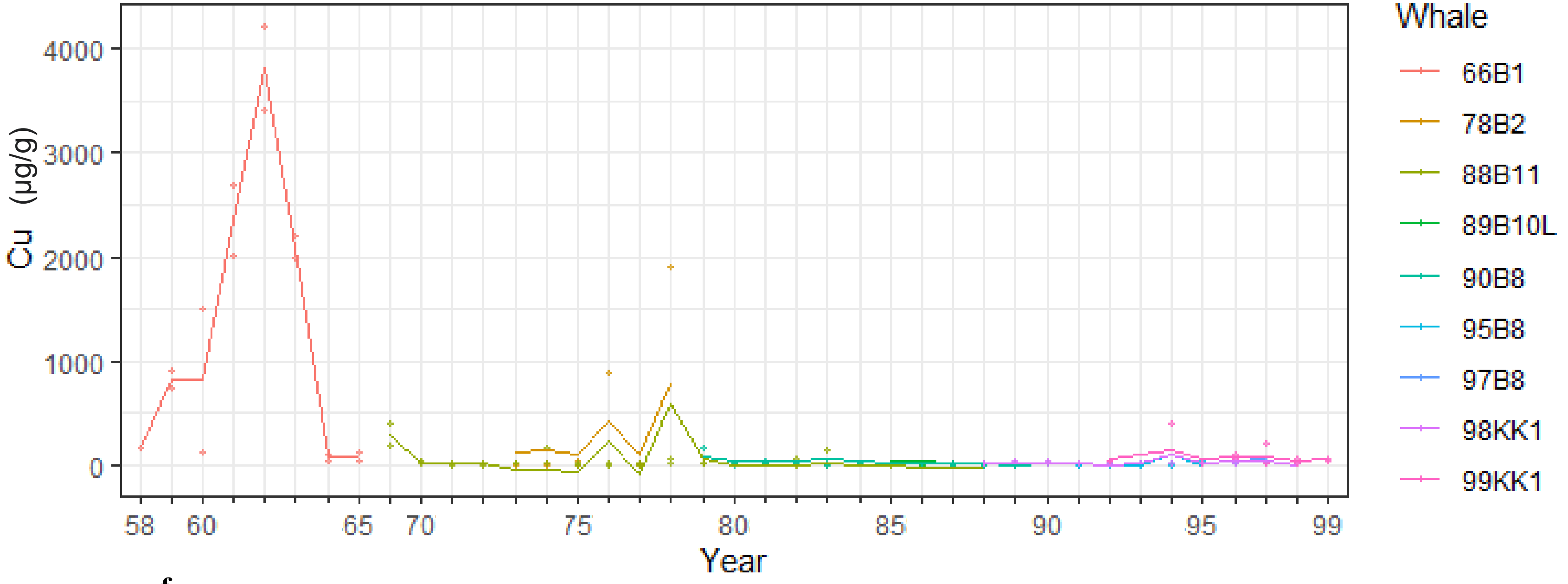
Cu (µg/g)
4000
3000
2000
1000
0
58
60
65
70
75
80
85
90
95
99
Year
Whale
66B1
78B2
88B11
89B10L
90B8
95B8
97B8
98KK1
99KK1

f

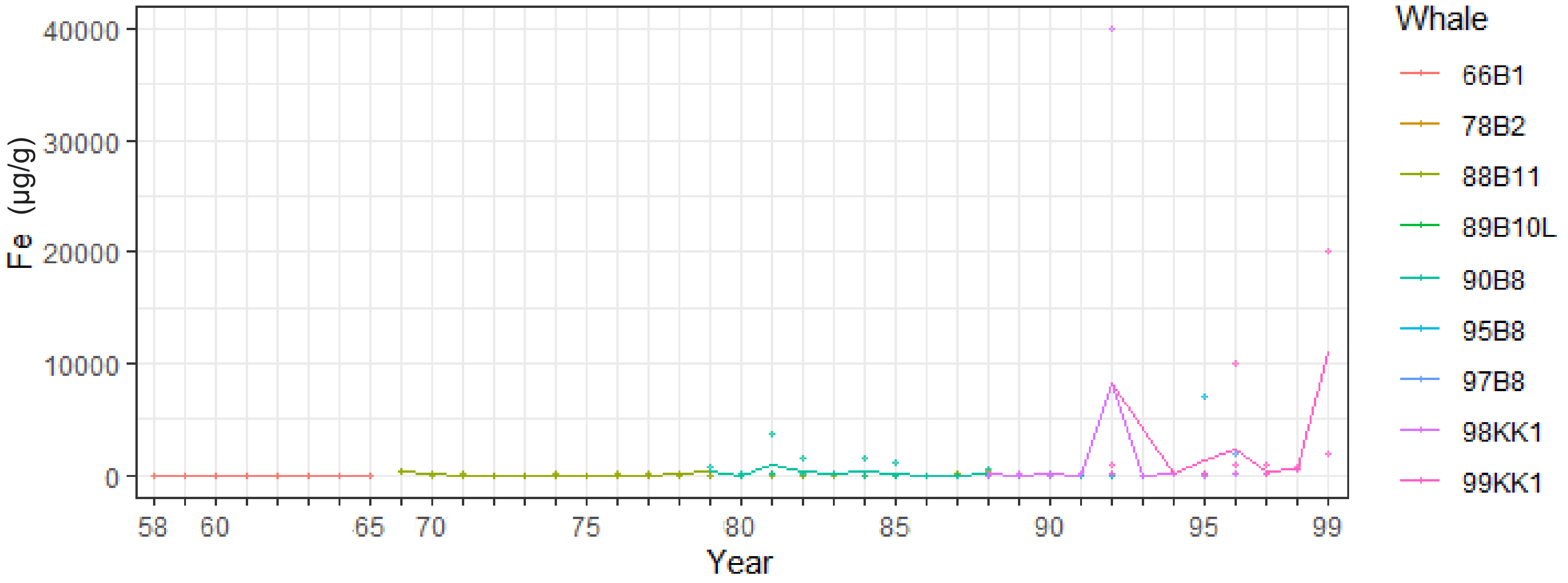
Fe (µg/g)
40000
30000
20000
10000
0
58
60
65
70
75
80
85
90
95
99
Year
Whale
66B1
78B2
88B11
89B10L
90B8
95B8
97B8
98KK1
99KK1

g

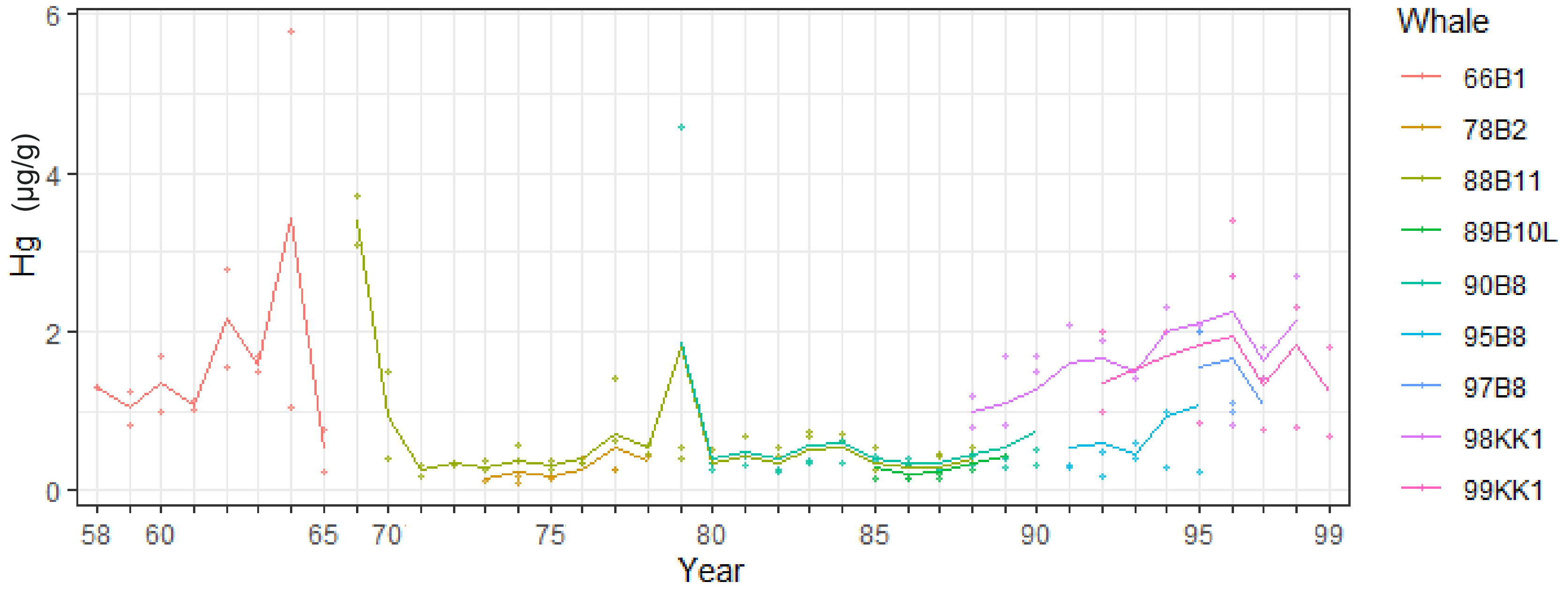

Hg (µg/g)
6
4
2
0
58
60
65
70
75
80
85
90
95
99
Year
Whale
66B1
78B2
88B11
89B10L
90B8
95B8
97B8
98KK1
99KK1

h

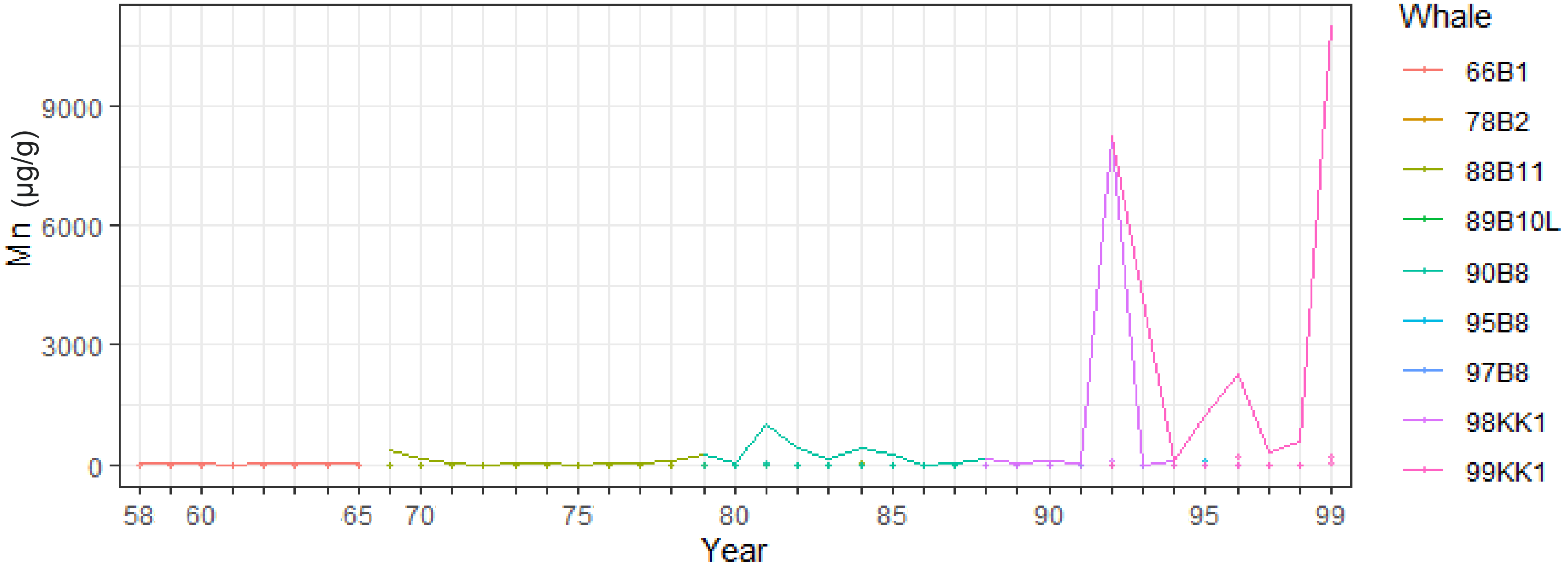

Mn (µg/g)
9000
6000
3000
0
58
60
65
70
75
80
85
90
95
99
Year
Whale
66B1
78B2
88B11
89B10L
90B8
95B8
97B8
98KK1
99KK1

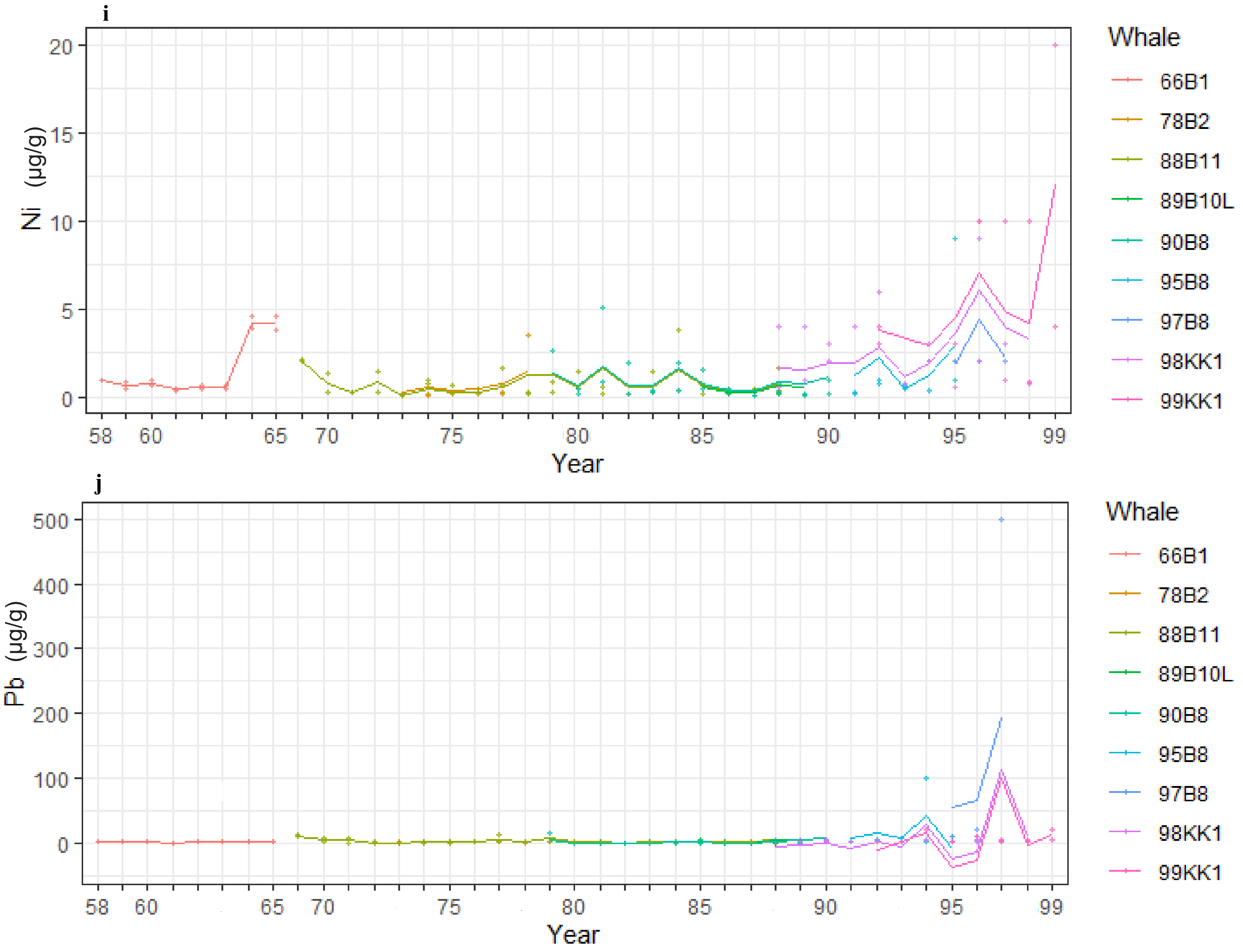

i
Ni (µg/g)
20
15
10
5
0
58 60 65 70 75 80 85 90 95 99
Year
Whale
66B1
78B2
88B11
89B10L
90B8
95B8
97B8
98KK1
99KK1
j
Pb (µg/g)
500
400
300
200
100
0
58 60 65 70 75 80 85 90 95 99
Year
Whale
66B1
78B2
88B11
89B10L
90B8
95B8
97B8
98KK1
99KK1

k

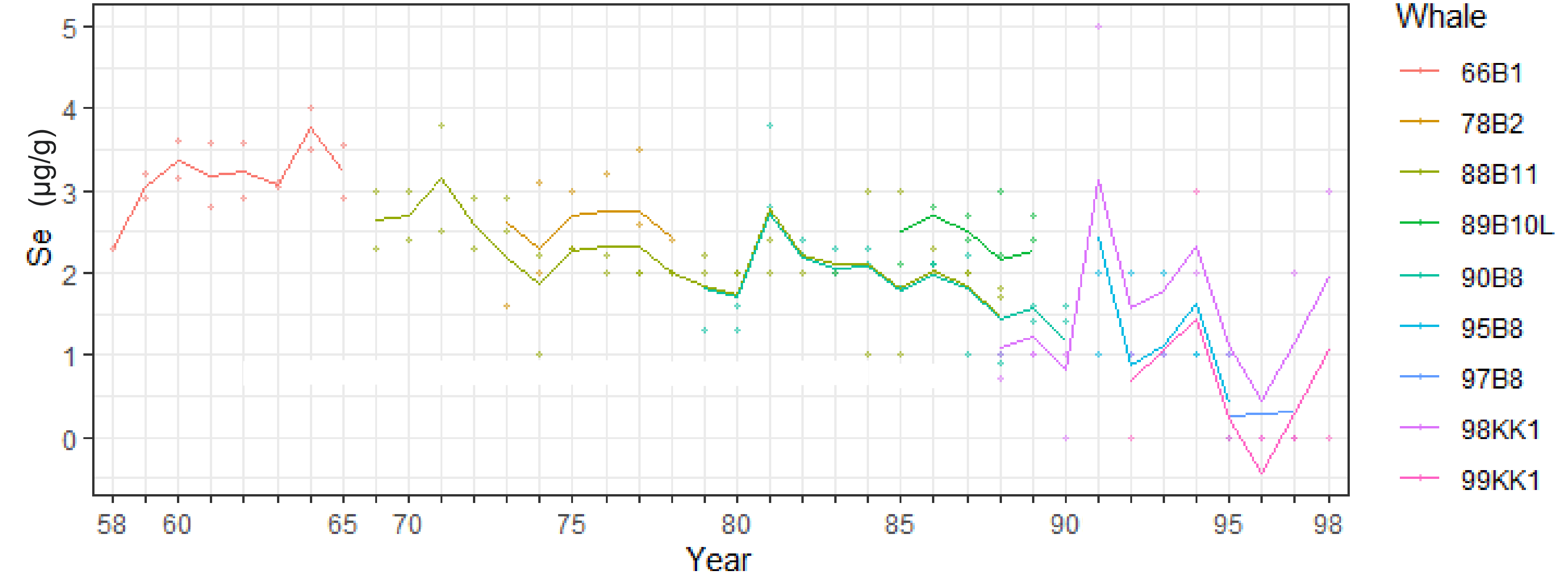
Se (µg/g)
0
1
2
3
4
5
58
60
65
70
75
80
85
90
95
98
Year
Whale
66B1
78B2
88B11
89B10L
90B8
95B8
97B8
98KK1
99KK1

l

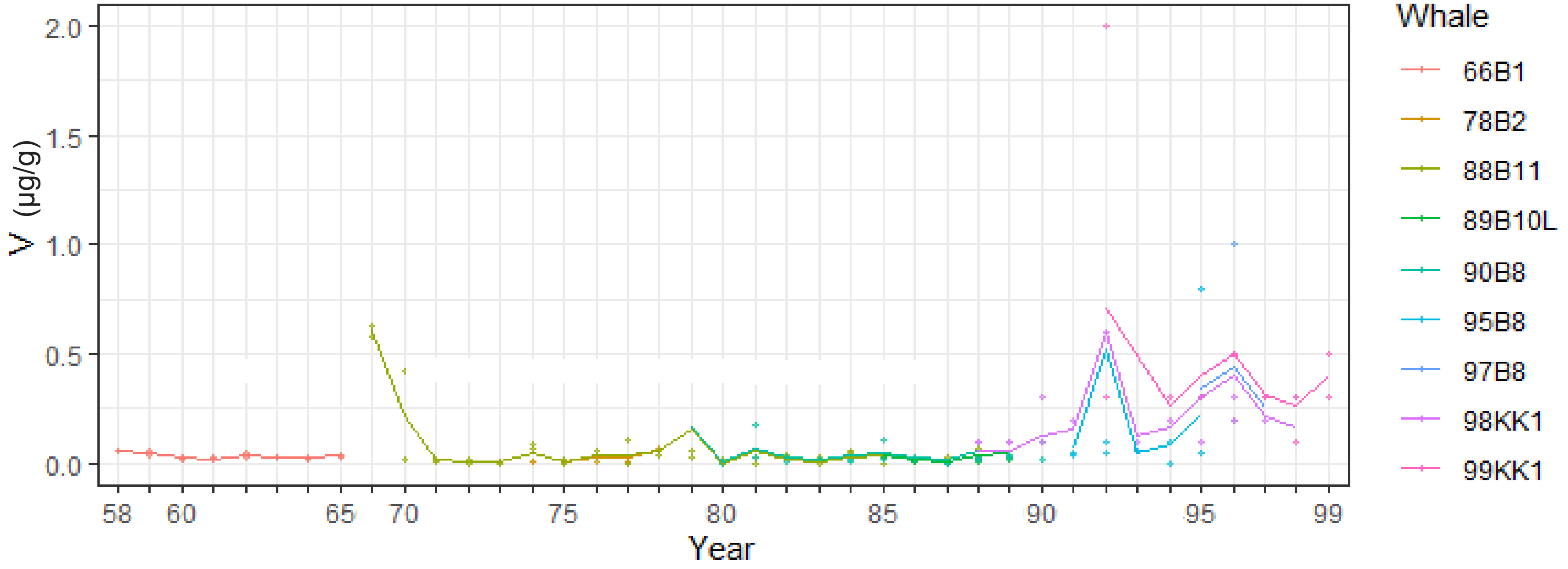
V (µg/g)
0.0
0.5
1.0
1.5
2.0
58
60
65
70
75
80
85
90
95
99
Year
Whale
66B1
78B2
88B11
89B10L
90B8
95B8
97B8
98KK1
99KK1

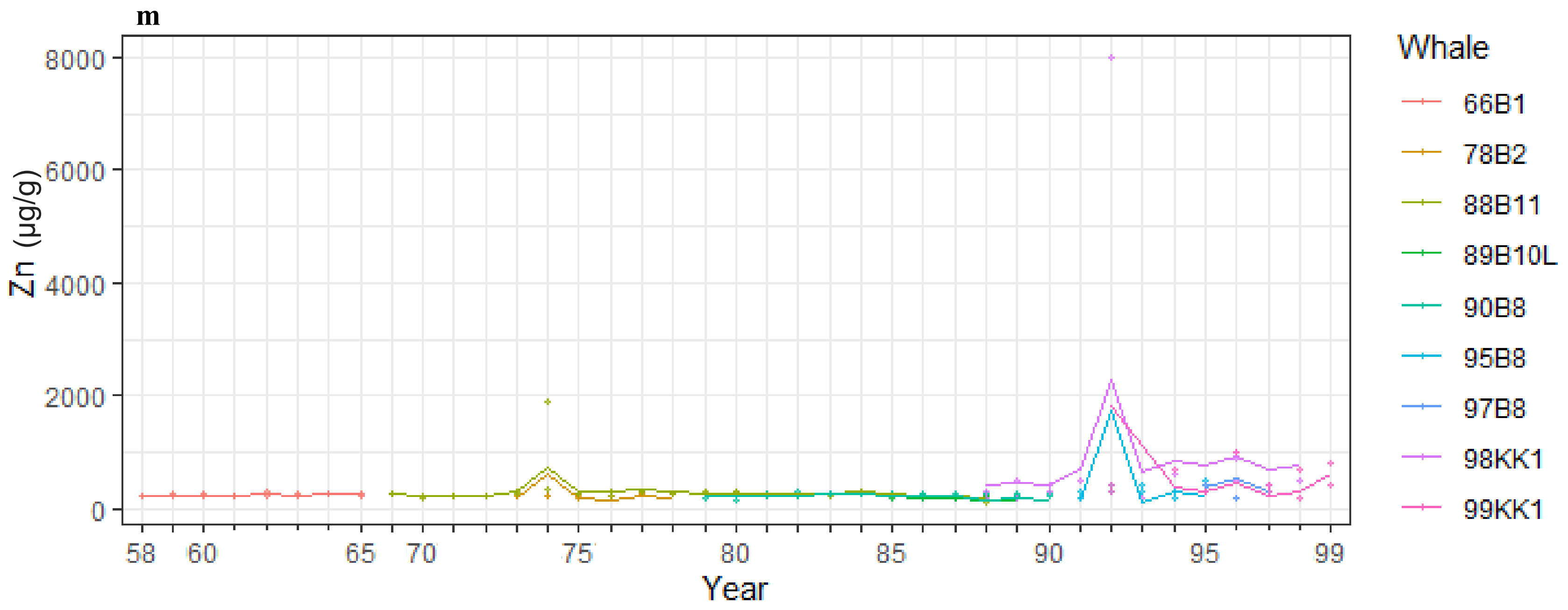

SM Figure 1 Concentrations (μg/g) of (a) Al, (b) Cd, (c) Co, (d) Cr, (e) Cu, (f) Fe, (g) Hg, (h) Mn, (i) Ni, (j) Pb, (k) Se, (l) V, (m) Zn from 1958–1999 (except 1967-1968). Data points represent detected concentrations, while regression lines illustrate trends per whale. All years represent the 1900s but are abbreviated to the last two digits of that year (ex: 1959 = "59" on x-axis).

SM Table 1 Individual whale parameters, including whale identification name (whale ID), sample size (n), date landed, location landed, sex, and age or age class [body length]. Whales were either attributed an age, age class, and/or body length.
*AK = Alaska
NA = not available
[+] L in whale ID name indicates a baleen plate from the whale's left side was taken
** = pregnant

Whale ID	n	Date Landed	Location Landed	Sex	Age [Age Class]	Body Length (m)
66B1	15	May 10, 1966	Barrow, AK*	Male	9 years [NA]	9.47
78B2	9	May, 1978	Barrow, AK	Male	4.5 years [subadult]	NA
88B11	40	Sept 17, 1988	Barrow, AK	Female	>22.5 years [NA]	NA
89B10L[+]	9	Oct 28, 1989	Barrow, AK	Female	NA [NA]	8.1
90B8	23	Oct 2, 1990	Barrow, AK	Male	>13.5 years [NA]	12.9
95B8	10	June 1, 1995	Barrow, AK	Female**	NA [NA]	15.2
97B8	5	May 15, 1997	Barrow, AK	Female	NA [adult]	NA
98KK1	15	Sept 4, 1998	Kaktovik, AK	Male	NA [subadult]	NA
99KK1	12	Sept 11, 1999	Kaktovik, AK	Female	NA [subadult]	NA
TOTAL	138					

SM Table 2 Detection limits (µg/g) of the ICP-MS for each element.

Element	Detection Limit (µg/g)
Al	0.1
As	0.00003
Cd	0.00001
Cr	0.0001
Co	0.00002
Cu	0.005
Fe	0.004
Pb	0.0004
Mn	0.00008
Hg	<0.00001
Ni	0.0005
Se	0.00003
Sn	0.001
V	0.00004
Zn	0.02

SM Table 3 Results of fitting a linear mixed-effects model to determine the importance of year, sex, and location when temporally analyzing concentrations of 13 elements in baleen of bowhead whales landed between 1966 and 1999 for element Al. Results for other elements provided upon request. The model chosen for Al (and all other elements) through AIC model selection has been bolded.
+ = data available per year and/or element upon request, but for simplicity of this table have been omitted

Model	Term	AIC	Coefficient	Standard Error
**Al ~ Year**	Intercept (Al)	**1534.753**	29.3	418.44
	Year+			
Al ~ 1	Intercept	2004.958	455.3918	180.9087
Al ~ Location	Intercept	1995.559	420.4330	182.6831
	Location		67.7904	53.6296
Al ~ Sex	Intercept	1993.210	407.5667	288.7033
	Sex		86.9976	387.9404
Al ~ Sex + Location	Intercept	1983.815	372.5831	289.4946
	Sex		87.1034	387.2178
	Location		67.7180	53.6281
Al ~ Sex * Location	Intercept	1973.500	338.8994	292.0167
	Sex		145.9229	392.2457
	Location		132.9274	81.9500
	Sex:Location		-113.9609	108.3328

SM Table 4 The best-fit linear mixed-effects models for each of the 13 elements tested, including each model's coefficient and standard error. All models had the same term of "Intercept (Element) ~ Year", where Element only included one element per model, not all 13 elements per model.

Model	Coefficient	Standard Error
Al ~ Year	29.3000	418.4403
Cd ~ Year	0.0451000	0.9943977
Co ~ Year	0.030000	2.219014
Cr ~ Year	0.030000	2.219014
Cu ~ Year	162.000	221.8008
Fe ~ Year	58.400	3960.198
Hg ~ Year	1.2900000	0.7542028
Mn ~ Year	0.69900	24.91512
Ni ~ Year	1.000000	2.314609
Pb ~ Year	2.34000	50.75064
Se ~ Year	2.3000000	1.0353807
V ~ Year	0.0530000	0.2030355
Zn ~ Year	0.0530000	727.8883

SM Table 5 Concentrations (µg/g) of all 13 elements (Al, Cd, Co, Cr, Cu, Fe, Hg, Mn, Ni, Pb, Se, V, Zn) per sample.

	Element Concentration (µg/g)												
Sample ID	Al	Cd	Co	Cr	Cu	Fe	Hg	Mn	Ni	Pb	Se	V	Zn
66B1-B1	15.7	0.0791	0.02	0.887	46.2	22.7	0.231	0.345	4.58	1.52	3.54	0.027	221
66B1-B2	33.7	0.061	0.23	0.899	109	23.1	5.79	0.42	3.85	1.52	4	0.03	262
66B1-B3	22	0.029	0.035	0.889	2200	20.6	1.69	0.313	0.614	0.977	3.04	0.029	206
66B1-B4	18.3	0.0381	0.033	1.46	3420	29.8	2.80	0.394	0.454	0.865	3.58	0.05	221
66B1-B5	26.2	0.033	0.046	0.746	2690	16.1	1.01	0.25	0.34	0.56	2.8	0.02	219
66B1-B6	37	0.046	0.02	0.67	130	17	1.0	0.37	0.904	1.32	3.6	0.02	230
66B1-B7	31.8	0.06	0.06	0.908	900	35.1	0.830	0.44	0.48	1.83	2.9	0.04	225
66B1-BC1	65	1	0.05	1.07	120	45	0.78	0.67	3.8	3.2	2.9	0.04	270
66B1-BC2	22.4	0.0849	0.049	1.08	42.8	24.7	1.05	0.361	4.61	2.55	3.51	0.02	267
66B1-BC3	49	0.043	0.02	2	2000	40	1.5	0.42	0.45	1.4	3.1	0.03	260
66B1-BC4	40.2	0.355	0.081	1.08	4220	29.3	1.54	0.37	0.649	1.53	2.9	0.03	290
66B1-BC5	10.9	0.186	0.783	0.9	2020	18.4	1.14	0.261	0.493	0.613	3.57	0.024	231
66B1-BC6	21.3	0.0521	0.045	0.93	1510	19.4	1.69	0.312	0.682	1.11	3.15	0.03	244
66B1-BC7	24.2	0.308	0.03	1.09	733	23.6	1.24	0.5	0.805	2.57	3.2	0.053	241
66B1-BC8	29.3	0.0451	0.03	1.27	162	58.4	1.29	0.699	1	2.34	2.3	0.053	211
78B2-B1	67	0.028	0.01	0.5	8.9	11	0.26	0.37	0.28	0.96	3.5	0.01	240
78B2-B2	16	0.019	0.052	2.2	890	39	0.39	0.54	0.2	1.3	3.2	0.01	210
78B2-B3	33	0.019	0.01	0.7	41	24	0.16	0.31	0.24	1.7	3	0.01	200
78B2-B4	17	0.026	0.01	0.16	6.89	11.2	0.10	0.26	0.085	0.836	3.1	0.01	214
78B2-BC1	55	N/D	0.08	18	1900	210	0.44	1.7	3.5	0.58	2.4	0.07	240
78B2-BC2	21	0.042	0.01	0.39	5.3	8.8	0.26	0.27	0.19	2	2.6	0.01	240
78B2-BC3	22.1	0.038	0.074	0.38	5.25	9.74	0.19	0.35	0.14	1.16	2.3	0.01	221
78B2-BC4	39.2	0.0515	0.02	0.39	4.85	16.4	0.17	0.39	0.21	2.13	2	0.01	230
78B2-BC5	33	0.065	0.01	0.22	5.1	31	0.11	0.32	0.19	0.98	1.6	0.01	210
88B11-B1	100	1.2	0.14	0.4	18	200	0.54	10	1.6	0.7	1.7	0.07	110
88B11-B2	150	0.053	0.03	0.74	7.1	91	0.45	2.8	0.5	0.92	2	0.03	230
88B11-B3	63	0.03	0.02	0.52	6.2	18	0.36	0.58	0.33	0.93	2.3	0.02	220
88B11-B4	75	0.022	0.03	0.65	5.3	16	0.26	0.39	0.18	0.46	3	0.03	210
88B11-B5	320	0.068	0.09	0.73	22	19	0.62	0.86	3.8	2.6	3	0.05	310
88B11-B6	180	0.02	0.03	0.61	6.1	11	0.68	0.37	0.26	0.96	2	0.03	230

88B11-B7	120	0.01	0.02	0.45	5.2	9.5	0.53	0.33	0.2	0.55	2.2	0.03	230
88B11-B8	92	0.023	0.02	0.81	11	17	0.43	0.34	0.16	0.42	2.4	0.03	230
88B11-B9	230	0.042	0.04	0.5	13	16	0.52	0.5	0.46	1.5	2	0	300
88B11-B10	160	0.065	0.03	0.62	16	16	0.53	0.47	0.82	1.2	2	0.03	300
88B11-B11	170	0.092	0.03	0.17	7.5	16	0.58	0.39	0.2	1.4	2	0.06	260
88B11-B12	240	0.125	2.2	0.9	10.5	19	0.62	0.6	0.65	13	2	0	280
88B11-B13	130	0.056	0.86	1.1	9	75	0.36	0.82	0.46	3.1	2.2	0.06	230
88B11-B14	120	0.027	0.04	0.5	13	13	0.37	0.31	0.67	2.2	2.3	0.02	250
88B11-B15	180	0.03	0.04	0.67	10	31	0.38	1.3	0.78	0.71	2.2	0.09	1900
88B11-B16	33	0.022	0.02	0.14	9.1	43	0.38	0.45	0.17	0.23	2.9	0.01	250
88B11-B17	52	N/D	0.04	4	10	12	0.33	0.52	1.4	0.21	2.9	0.02	230
88B11-B18	48	0.01	0.02	0.35	5.9	80	0.19	0.8	0.28	0.3	3.8	0.02	220
88B11-B19	69	0.047	0.63	0.39	6.7	20	0.39	0.42	0.22	2.2	3	0.02	200
88B11-B20	270	0.673	0.37	1.8	190	380	3.7	5.9	2	13	3	0.63	250
88B11-BC1	110	0.027	0.05	0.32	6.1	340	0.36	4.1	0.32	0.69	1.8	0.03	210
88B11-BC2	100	0.025	0.02	0.29	4.6	200	0.42	1.4	0.4	0.75	2	0	220
88B11-BC3	130	0.076	0.02	0.79	14	16	0.31	2.2	0.31	1.5	2	0.02	220
88B11-BC4	260	0.04	0.05	1.4	9.5	24	0.55	1.4	0.64	2.1	1	0	260
88B11-BC5	300	0.061	0.06	2	10	30	0.7	30	0.4	2	1	0.06	300
88B11-BC6	310	0.02	0.05	1.5	140	260	0.73	2.4	1.4	2.4	2	0	270
88B11-BC7	220	0.01	0.02	1.3	66	37	0.42	0.5	0.2	0.43	2	0.04	220
88B11-BC8	200	0.02	0.08	0.71	20	95	0.67	0.96	0.54	1	2	0	270
88B11-BC9	160	0.02	0.02	0.33	18	38	0.35	0.57	1.4	1.2	2	0.02	280
88B11-BC10	94	0.061	0.03	0.34	6.9	23	0.39	0.94	0.31	1.6	2.2	0.06	240
88B11-BC11	120	0.026	0.04	0.41	51	27	0.45	7.5	0.22	1.7	2	0.04	270
88B11-BC12	180	0.089	0.07	1.4	6.4	77	1.4	1.3	1.6	2.7	2	0.11	290
88B11-BC13	160	0.02	0.05	0.5	6	16	0.34	0.33	0.25	0.99	2	0.03	280
88B11-BC14	79	0.018	0.04	0.1	5.1	14	0.27	0.27	0.26	0.53	2.3	0	240
88B11-BC15	240	0.046	0.07	0.96	160	160	0.56	1.3	1	2.62	1	0.07	340
88B11-BC16	20	0.017	0	0.1	8.4	8.11	0.26	0.19	0.1	0.171	2.5	0	300
88B11-BC17	43	0.014	0.01	0.21	6	11	0.34	0.24	0.28	0.89	2.3	0	230
88B11-BC18	52	0.032	0.02	0.24	14	26	0.31	0.31	0.27	7.1	2.5	0.01	230
88B11-BC19	120	0.36	0.25	0.95	46	240	1.5	3.4	1.3	6.5	2.4	0.42	210
88B11-BC20	230	0.49	0.36	1.5	390	310	3.1	4.3	2.1	9.5	2.3	0.58	240
89B10L-B1	45	0.031	0.02	0.27	8	42	0.44	0.75	0.19	0.943	2.7	0.03	220

89B10L-B2	25	0.011	0.03	0.13	4.9	8	0.45	0.47	0.23	0.617	3	0.01	210
89B10L-B3	45	0.014	0.01	0.24	9.8	8.4	0.22	0.28	0.41	0.516	2.7	0.01	180
89B10L-B4	28	0.023	0.02	0.19	6.5	5.7	0.14	0.25	0.24	0.641	2.8	0.01	180
89B10L-B5	110	0.21	0.85	0.46	15	29	0.15	0.78	0.64	5.09	2.1	0.03	190
89B10L-BC1	31	0.015	0.02	0.26	5.9	11	0.44	0.33	0.15	1.3	2.4	0.02	200
89B10L-BC2	30	0.013	0.02	0.21	6.3	5	0.25	0.25	0.17	0.38	2.2	0.02	200
89B10L-BC3	29	0.0088	0.01	0.16	6.3	7.2	0.15	0.27	0.26	0.44	2.4	0.01	200
89B10L-BC4	42	0.026	0.03	0.24	8.1	7.7	0.15	0.31	0.19	0.53	2.1	0.01	200
90B8-B1	84	0.42	0.14	1.3	14	210	0.32	2.5	0.96	4.4	1.4	0.1	250
90B8-B2	66	0.018	0.05	0.51	3.1	42	0.41	0.34	0.19	0.2	1.6	0.04	240
90B8-B3	89	0.08	0.06	0.45	4.8	530	0.32	3.3	0.73	1.2	1	0.02	250
90B8-B4	110	0.042	0.02	0.1	4.4	10	0.25	0.24	0.1	0.64	1	0	240
90B8-B5	75	0.018	0.01	0.55	13	14	0.31	0.24	0.3	0.37	2.1	0.02	250
90B8-B6	77	0.03	0.04	1.73	7.3	45	0.43	0.56	0.42	0.62	1.8	0.02	250
90B8-B7	73	0.024	0.01	0.24	11	9.5	0.34	0.21	0.34	0.67	2.1	0.01	240
90B8-B8	38	0.024	0.03	0.25	7.5	210	0.35	1.4	0.32	0.22	2.3	0.02	250
90B8-B9	60	0.032	0.14	1.21	8.2	1500	0.23	9.6	1.9	0.51	2.4	0.03	280
90B8-B10	69	0.035	0.38	3.52	39	3800	0.44	25	5.1	1.2	3.8	0.18	250
90B8-B11	48	0.015	0.02	2.12	10	110	0.34	0.92	0.47	0.35	1.6	0.01	210
90B8-B12	160	0.84	0.51	2.04	170	810	4.59	8.6	2.6	15	1.3	0.39	200
90B8-BC1	61	0.044	0.47	0.27	12	19	0.51	0.38	0.14	1.7	1.6	0.02	230
90B8-BC2	69	0.014	0	0.17	5.1	15	0.29	0.27	0.1	0.35	1.4	0.03	230
90B8-BC3	210	0.03	0.03	0.47	6.6	17	0.43	0.3	0.31	1.5	0.9	0.03	240
90B8-BC4	76	0.022	0.01	0.17	6.9	10	0.25	0.27	0.1	0.33	2.2	0	230
90B8-BC5	100	0.01	0.06	0.45	13	40	0.40	0.47	0.44	1.1	2.1	0.03	250
90B8-BC6	90	0.078	0.14	1.43	27	1100	0.37	7	1.5	3.1	1.8	0.11	250
90B8-BC7	47	0.011	0.14	1.1	9.9	1600	0.62	10	1.9	0.56	2.3	0.02	260
90B8-BC8	32	0.13	0.03	0.23	5.6	230	0.37	1.7	0.3	0.9	2	0.02	240
90B8-BC9	53	0.0082	0.01	0.22	8.5	78	0.27	0.7	0.2	0.45	2.2	0.01	220
90B8-BC10	61	0.018	0.06	1.32	11	180	0.32	1.5	0.82	0.37	2.8	0.03	230
90B8-BC11	43	0.0089	0.01	0.13	5	35	0.25	0.34	0.13	0.41	1.3	0	150
95B8-B1	1000	0.4	7	5	30	7000	2	80	9	9	0	0.8	500
95B8-B2	700	2	4	3	20	80	1	2	2	100	1	0.1	300
95B8-B3	300	0.1	2.1	0.4	9	20	0.4	0.8	0.7	7	2	0.06	300
95B8-B4	200	0.25	0.05	0.85	6	31	0.18	0.94	0.8	3.8	2	0.05	400

95B8-B5	200	0.02	0	0.89	6	16	0.31	0.44	0.2	1.1	2	0.04	200
95B8-BC1	240	0.095	0.2	0.9	11	23	0.24	0.79	1	1.9	1	0.05	300
95B8-BC2	210	0.04	0.05	0.3	6	73	0.29	0.91	0.4	1.1	1	0	200
95B8-BC3	300	0.04	0.06	0.8	6	30	0.6	0.7	0.5	2	1	0.06	400
95B8-BC4	500	0.04	0	1	10	50	0.5	1	1	2	1	0.1	300
95B8-BC5	300	0.04	0.05	1.8	6.4	30	0.28	1.3	0.3	1.7	1	0.05	300
97B8-B1	800	0.1	0.2	8	20	2000	1	10	2	5	0	0.2	200
97B8-B2	1000	0.2	0.3	20	20	200	2	3	2	10	0	0.3	400
97B8-BC1	600	10	20	6	30	100	1.4	1	2	500	0	0.3	300
97B8-BC2	2000	0.3	5	6	20	200	1.1	2	2	20	0	1	200
97B8-BC3	1000	0.2	0.3	6	20	100	2	2	2	9	0	0.3	400
98KK1-B1	800	0.07	0	4	20	100	1.8	2	3	2	2	0.2	400
98KK1-B2	1000	0.2	0.2	3	20	100	0.82	6	2	3	0	0.2	500
98KK1-B3	1000	0.6	3.2	4	20	100	2.3	2	3	6	2	0.2	600
98KK1-B4	700	0.1	2.2	30	20	40000	1.9	100	6	2	1	0.6	8000
98KK1-B5	600	0.08	0.9	4	10	60	1.5	0.9	2	2	0	0.1	300
98KK1-B6	800	0.1	0.8	5	30	90	1.7	2	4	4	1	0.1	500
98KK1-B7	600	0.01	0	2	10	50	1.2	0.6	0.6	0.9	1	0.1	200
98KK1-BC1	1000	0.05	0	3	20	500	2.7	7	0.8	2	3	0.3	500
98KK1-BC2	2000	0.2	0.5	6	60	200	2.7	3	9	9	0	0.3	900
98KK1-BC3	700	0.03	0	3	10	50	2.1	0.6	0.6	1	1	0.1	300
98KK1-BC4	400	0.02	0	1	10	0	1.4	0.7	0.8	0.9	1	0.1	200
98KK1-BC5	1000	0.08	0.2	7	20	100	2.1	4	4	3	5	0.2	500
98KK1-BC6	1000	0.1	0.5	4	30	70	1.7	1	3	4	1	0.3	400
98KK1-BC7	400	0.02	0.1	2	20	60	0.83	0.9	1	1	1	0.1	200
98KK1-BC8	400	0.2	0.1	14	10	80	0.81	1	4	6	0.7	0.1	300
99KK1-B1	1000	1	2	30	70	20000	1.8	200	20	20	0	0.5	800
99KK1-B2	500	0.04	0.1	2	10	700	0.79	6	0.9	1	0	0.1	200
99KK1-B3	800	0.1	0	2	20	100	0.78	3	1	3	0	0.3	300
99KK1-B4	3000	1	0.5	20	100	10000	2.7	200	10	10	0	0.5	1000
99KK1-B5	700	0.2	0.1	10	20	200	0.85	3	3	1	0	0.3	300
99KK1-B6	1000	0.05	0.5	10	30	1000	2.0	10	4	1	0	2	300
99KK1-BC1	2000	0.3	0.3	6	40	2000	0.67	20	4	6	0	0.3	400
99KK1-BC2	2000	0.3	0.5	7	70	600	2.3	8	10	4	0	0.3	700
99KK1-BC3	900	0.1	0.3	2	200	1000	1.4	10	10	5	0	0.3	400

99KK1-BC4	3000	0.4	0.5	10	50	1000	3.4	10	10	9	0	0.5	1000
99KK1-BC5	1000	0.1	0	7	400	100	2.0	2	2	20	3	0.3	700
99KK1-BC6	2000	0.08	0	10	40	200	1.0	3	3	2	0	0.3	400
